# Supplementary material for: Large language models approach expert-level clinical knowledge and reasoning in ophthalmology: A head-to-head cross-sectional study
Source: PLOS Digit Health. 2024 Apr 17;3(4):e0000341. doi: 10.1371/journal.pdig.0000341 (PMC11023493; doi:10.1371/journal.pdig.0000341)
Supplement: S1 Protocol — (PDF) [file pdig.0000341.s006.pdf]

S1 Protocol

Premise

Large language models are causing great excitement across fields including medicine, particularly with the general release of ChatGPT. The older version of ChatGPT (powered by GPT-3.5) attained pass level performance in the USMLE, and newer versions powered by GPT-4 have attained expert level performance. Here, we want to trial the potential for ChatGPT in ophthalmology by testing it on the FRCOphth Part 2 MCQ, which all ophthalmologists in the UK must pass to complete training and practice independently. Darren Ting, study supervisor, has published a book of practice questions for the FRCOpth Part 2, which serves as a convenient resource for this trial. His book features ~360 questions testing theoretical and clinical knowledge.

The team has three tasks: to answer 90 examination questions; grade ChatGPT's accuracy and relevance; and indicate which of two responses is preferred overall.

Question answering

For every question in Chapter 6 (90 questions in total) of the textbook, an answer should be inputted into the second column of the spreadsheet (Figure 1). There should be no referring to answers given later in the textbook, other resources, or ChatGPT outputs—our aim is to gauge the usual performance of ophthalmologists who have previously passed the examination.

Question	Answer
1	A
2	C
3	D
4	A
5	A
6	D
7	C
8	D
9	

Figure 1 | Exemplar data entries for the question answering task (answers made up randomly!).

Grading accuracy and relevance; and indicating preference

To gauge accuracy and relevance, Likert scales are employed ranging from 1-5: (1) = very bad, (2) = bad, (3) = acceptable, (4) = good, (5) = very good. These are subjective, qualitative metrics—answer as best as possible with consistency across the 90 questions, as if an ophthalmology trainee were giving answers to the questions. To assist grading, the question text and correct answer will be provided alongside the responses (Figure 2).

For each question, two responses will be provided. The order of presentation of GPT-3.5 and GPT-4 output will be random. After grading each response 1-5 for accuracy and relevance, the final task is to indicate which of the two responses you prefer—which is the best response to the question asked?

question	category	correct answer	output 1	answer 1	Accuracy	Relevance	output 2	answer 2	Accuracy 2	Relevance 2	Preferred response
52. Which of the following features of choroidal naevus is associated with increased risk of transformation to choroidal melanoma? A. Absence of ultrasonographic hollowness B. Absence of halo C. Tumour thickness of 1 mm D. Presence of drusen	uveitis and oncology	B	C. Tumour thickness of 1 mm<br><br>Increased tumor thickness is one of the factors associated with a higher risk of transformation from choroidal nevus to choroidal melanoma. A tumor thickness of 2 mm or more is generally considered a significant risk factor, but even a thickness of 1 mm can be associated with an increased risk. Other factors that may increase the risk of transformation include the presence of subretinal fluid, ultrasonographic hollowness, orange pigment, and absence of a surrounding halo.	C	2	5	[illegible] of choroidal naevus that is associated with an increased risk of transformation to choroidal melanoma.<br><br>Choroidal naevus is a benign tumor of the eye that arises from the melanocytes of the choroid layer. In some cases, it may transform into a choroidal melanoma, which is a malignant tumor that can be life-threatening. Therefore, it is important to identify the features of choroidal naevus that are associated with an increased risk of transformation.	B	1	4	1

Figure 2—Exemplar responses for the grading and preference tasks. Columns highlighted red require input; all other data are provided.

The second data sheet will be provided upon completion of the first task, to facilitate proper blinding. Please liaise with Arun **[REDACTED]** to receive data sheets, the textbook, return completed data sheets, or solve any issues that arise.

Many thanks for your involvement, and best of luck!

Arun Thirunavukarasu and Darren Ting
